# 

**Published:** 1873-01-15

**Authors:** 


New York State Medical Society.—The next regular annual meeting of the Medical Society, of the State of New York, will be held at Albany, commencing Feb. 4th, and continuing three days. This is one of the oldest and most efficient State medical societies in the country, and the coming meeting will be one of interest.

MONEY RECEIPTS.—We acknowledge remittances received from Drs. L. D. Humphrey, J. M. Cole, C. O'Brien, W. L. Kreider, A. B. McCandless, F. Merriman, D. C. Roundy, E. W. Capron, J. R. Doig, A. L. Bartlett, H. H. Clark, G. W. Morrill, L. B. Cowles, J. H. Smith, I. N. McLane, V. H. Coffman, J. R. Webster, B. R. Helmes, J. F. Kelsey, W J. Fairman.—Jan. 8th, 1873.

